# Rapid adsorption enthalpy surface sampling (RAESS) to characterize nanoporous materials†

**DOI:** 10.1039/d2sc05810c

**Published:** 2023-01-17

**Authors:** Emmanuel Ren, François-Xavier Coudert

**Affiliations:** a CEA, DES, ISEC, DMRC, Univ. Montpellier Marcoule France; b Chimie ParisTech, PSL University, CNRS, Institut de Recherche de Chimie Paris 75005 Paris France fx.coudert@chimieparistech.psl.eu

## Abstract

Molecular adsorption in nanoporous materials has many large-scale industrial applications ranging from separation to storage. To design the best materials, computational simulations are key to guiding the experimentation and engineering processes. Because nanoporous materials exist in a plethora of forms, we need to speed up the existing simulation tools to be able to screen databases of hundreds of thousands of structures. Here, we describe a new algorithm that quickly calculates adsorption enthalpies by sampling the surface of the material instead of the whole porous space. This surface sampling has been tested on the CoRE MOF 2019 database and has been proven to be more than 2 orders of magnitude faster than the gold standard method (Widom insertion), with an acceptable level of error on an enthalpy value of 0.34 kJ mol^−1^, and is therefore proposed as a valuable addition to the high-throughput screening toolbox.

## Abstract 2

To speed up the calculation of adsorption enthalpy, a new algorithm changes the 3D problem into 2D sampling. It reduces the computational cost of large-scale screening of nanoporous materials for applications like fluid separation and storage.

## Graphical abstract

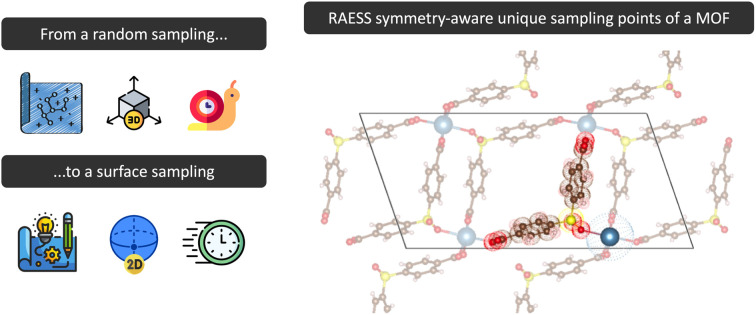

## 1 Introduction

Molecular adsorption has many large-scale industrial applications in our society, including fluid storage, molecular separation, and purification, and is therefore a very active area of research in both chemistry and materials science. Improvement in the performance of adsorption-based processes could reduce the environmental impact of separation and purification applications by replacing energy-intensive processes such as cryogenic distillation.^1,2^ In the energy industry, the use of nanoporous solids is a promising low-energy alternative to pressurized gas tanks for the storage of fuel such as H_2_ or CH_4_.^3^ Adsorption is also foreseen as a crucial component in the next generation of carbon capture and storage systems.^4^ To further unfold the potential of this technology, the design of materials for targeted applications needs to be refined.

Although the final steps of this design process can only be achieved by experimentation and engineering, computational simulations can play a key role in guiding the design process and speeding up the discovery of materials for targeted applications. Thanks to initiatives such as the Materials Genome project,^5,6^ we now have databases of hundreds of thousands of structures at our disposal, including both experimentally known and theoretically predicted structures, along with their associated physical and chemical properties. These databases can be computationally screened to retrieve key structure–property relationships, potential theoretical limitations and promising new structures.^7,8^ This is particularly true in the case of nanoporous materials, which have been extensively studied in high-throughput screening methodologies^9^ for the identification of top-performing materials for applications such as electrocatalysis,^10^ photocatalysis,^11^ heterogeneous catalysis,^12^ membrane separation,^13^ adsorptive separation,^14^ adsorptive storage,^15^ mechanical behaviors,^16^*etc.*

Because high-throughput screening is performed on the scale of hundreds of thousands or even millions of structures, there is a need for faster computational methods to predict the materials' properties, to be able to analyze larger and larger databases. In the field of adsorption, several measures have been proposed in order to study the performance of a material for the adsorption of a specific guest molecule. The most accurate, but also the most computationally expensive one, is the grand canonical Monte Carlo (GCMC) simulation. On the other end of the scale, the use of simple geometric descriptors (pore size, pore volume, and surface area) has also been proposed as proxies for various adsorption-related metrics. As the field of computational chemistry is turning more and more towards machine learning (ML), the development of a wide range of rapidly calculable descriptors^17^ is an exciting research focus especially to speed up computational screenings.

For low-pressure adsorption, a widely used characterization parameter is the adsorption enthalpy Δ*H*_ads_, which describes the affinity of an adsorbate molecule with the internal surface of a porous framework. In theory,^18^ this value is derived from the continuous Boltzmann average of the interaction energies *E*_int_ of the adsorbate with the framework over the entire porous space, and this integral is calculated using a discrete summation over a set of well-chosen points *i* (note that the −*RT* term comes from the ideal gas approximation):1
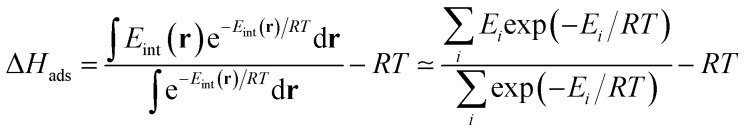


However, in molecular simulations, a complete sampling of the free volume can be extremely cumbersome. Therefore, random samplings are used to reduce simulation time, for example in the Widom insertion method.^19^ Still, convergence of the random sampling of space requires a large number of samples, and most of the points may not contribute significantly to the Boltzmann average (*i.e.*, they might have high energy). It is possible to reduce the computational cost further by reducing the number of sampled points, to try and capture only those with the highest contribution to the actual value of enthalpy. But, how can we choose these points *a priori*, without first calculating their energy?

One such biased sampling methodology was recently proposed and used in a computational screening study of adsorptive separation of xenon from krypton. Simon *et al.* used a machine learning model to screen over 670 000 structures based on geometrical descriptors and one energy descriptor that mainly explains the accuracy of the final model. To calculate this descriptor, the authors used the average of the interaction energy over the Voronoi network to account for the exponential contribution of the energy in the Henry constant. According to the authors, this approach can sample the most favorable sites of the structure without wasting computational time on unfavorable ones. However, because the sampled points are at the center of the cages, they may not always be the most attractive sites especially for large pores. This approximate approach makes this sampling interesting for quickly producing an energy-based ML descriptor, but it could not replace a Widom insertion for assessing the adsorption performance. Other biased sampling methods that calculate the integrals of the adsorption enthalpy and the Henry constant could be applied, instead. In this work we propose one way of exploiting prior chemical knowledge of adsorption—the fact that it occurs near the surface of the pores—to efficiently sample the nanoporous space.

We propose a novel algorithm for rapid adsorption enthalpy characterization, based on the reduction of the sampling space from 3D to 2D. This method is more accurate than the previous Voronoi sampling strategy. Moreover, the dimension reduction coupled with symmetric properties makes it faster than the standard Widom insertion method. This algorithm has been tested on xenon and is directly applicable to any spherical adsorbate model; and it can then be adapted to polyatomic adsorbate molecules as well. This algorithm can be used in the future to speed up the calculation of adsorption properties in regular or ML-assisted high-throughput computational screenings of nanoporous materials.

## 2 Methods

### 2.1 Benchmark

Before describing the core components of our surface sampling algorithm, we briefly present the other simulation tools used in the article, for comparison and benchmarking purposes. We used the RASPA2 software for calculations such as Widom insertion and surface area calculations.^20^ For the Voronoi sampling technique, we used the Zeo++ software to determine the positions of the Voronoi nodes.^21^ All the calculations were performed by considering the CoRE MOF 2019 (ref. 22) structures as rigid. In order to speed up the surface sampling, our algorithm exploits the symmetry of the material, looping over all symmetry-inequivalent atoms. Since the space groups (and symmetry operations) of the nanoporous structures in the CoRE MOF database were not specified, we used a Python script based on the Spglib library to determine them.^23^ The unique atoms are defined according to the symmetry determined by the aforementioned Python script.

All simulations are performed for xenon adsorption on structures of the CoRE MOF 2019 database at infinite dilution and at 298 K. Krypton adsorption has also been considered in order to see the viability of the method in prediction of Xe/Kr selective materials (see full details in ESI† Section S4). Adsorption at another temperature (600 K) was tested using the final algorithm, and the results are presented in ESI† Section S2. Other databases have also been briefly explored to test the robustness of the proposed algorithm. A subset of a hypothetical MOF database, the ToBaCCo database,^24^ has been screened (Section S6.1†), because it contains very different MOFs according to the diversity analysis of Moosavi *et al.*^25^ (for instance, the pores are larger). An amorphous material database (containing 205 structures) has also been screened and the algorithm identified some top materials for xenon adsorption (Section S6.2†).

Widom insertion^19^ is implemented in RASPA2. It consists in randomly inserting a single molecule inside an existing molecular system to measure an interaction energy. In adsorption simulations, these interaction energies of the randomly inserted adsorbate are typically used to determine values of the Henry constant *K*_H_ and of adsorption enthalpy Δ*H*_ads_ at the zero-loading limit.

The surface area calculation implemented in RASPA2 follows a very standard “rolling ball” algorithm^26^ based on hard spheres with sizes determined by the sigma value of the Lennard-Jones potential. First, a probe-molecule samples the spherical surface of the atoms of the framework. A portion of the sphere is excluded, because the probe overlaps with another atom. Each framework atom has an area of adsorbable surface, and the sum of all the areas gives the total adsorbable surface area. The values given by these simulations are then compared to the values given by our algorithm to check the consistency of our implementation.

In mathematics, a tessellation of a given space corresponds to a partition into non overlapping sub-spaces. In the Voronoi tessellation, named after Georgy Feodosevich Voronoy 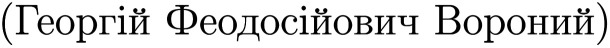, a set of points (seeds) are associated with a tessellation of regions (Voronoi cells) so that each seed has a cell that is closer to it than any other seeds.^27^ Applied in materials science, the Voronoi cells associated with each atom of the framework can be used to determine key geometrical descriptors (void volume, accessible surface area, and pore sizes). At the vertices of each cell, there are Voronoi nodes that were used in the Voronoi energy calculation presented by Simon *et al.*^14^ To compare our algorithm to a Voronoi sampling, we used the interaction energy values at the Voronoi nodes to calculate a Boltzmann average. These proxies for the adsorption enthalpy are then indirectly compared to the adsorption enthalpies calculated by our surface sampling.

### 2.2 Force field

To model the van der Waals interactions, we used Lennard-Jones (LJ) truncated and shifted potentials with a 12 Å cut-off without tail corrections. The atoms of the structures were modeled using the LJ parameters from the universal force field UFF.^28^ For xenon we used the following LJ parameters:^29^*ε*_Xe_ = 221.0 K and *σ*_Xe_ = 4.100 Å.^30^ To determine cross interaction parameters between xenon and the host atoms, we used the Lorentz–Berthelot combination rules.^31^ Throughout the article, all interaction energies are calculated with these same parameters, and while the exact results in terms of adsorption enthalpies depend on the force field chosen, the goal of this article is the comparison of different methodologies. We want to stress that the sampling method proposed herein could be used with any other force field. Other analytical forms could be used, or other LJ parameters could be chosen; for example, it is standard to mix Dreiding^32^ for the organic part and UFF for the inorganic part of the MOF structures.

### 2.3 Simulation box

To design a versatile simulation tool, we use periodic boundary conditions to create a rectangular simulation box for structures with non-rectangular unit cells. An extended neighbor list is created from the atoms of the translated rectangular boxes within the chosen cutoff. When looping over the unique atoms of the framework in the surface sampling, this neighbor list is restrained to a shorter neighbor list to be used in the interaction energy calculation, like in most molecular simulation algorithms.^33^ To evaluate the effect of the neighbor list, we tested the final implementation without the implementation of the neighbor list, and the simulation ran for 12.6 s instead of 0.34 s (37 times slower) without altering the accuracy. This shows that the implementation of an efficient neighbor list is a key point in our algorithm.

### 2.4 Sphere sampling algorithm

In our proposed method as in the surface area calculation algorithm,^26,34^ we rely heavily on the use of a uniform sampling of *n* points on the surface of a sphere, but this problem can be quite challenging in and of itself. In fact, except for very specific values of *n*, there is not a general analytical solution to the problem, only numerical approximations. During the development of our algorithm, we tested several existing methodologies^35^ to achieve this sampling.

The first technique is to rely on random sampling, with no guarantee of uniformity, but which should converge for a large *n*. To generate random 3D unit vectors, one approach is to draw random vectors in the corresponding cube, rejecting the points that are not inside the sphere. A simple normalization of the remaining vectors gives a random sampling of the sphere surface.^36^ The same result can also be achieved by generating three normally distributed random values and normalizing the vector obtained by these numbers.^36,37^

Another technique to obtain a uniform distribution is to imagine using a simulation of *n* charged points on the surface of a sphere and minimize their electrostatic repulsion. This method, based on the Thomson problem,^38^ relies on numerical optimization and can become very expensive if *n* is high.^39^

The method we found to be the most efficient (slightly faster) for the typical values of *n* that we consider (between 100 and 300 000) consists in wrapping a string of points around the sphere in a spiral manner. This technique is closer to laying a uniform grid over the surface than a “random sampling” of the surface, which avoids redundant sampling points. The height *h* = *r* cos *ϕ* (where (*r*, *θ*, *ϕ*) are the spherical coordinates) of the sphere is uniformly divided into *n* points; for each of these heights we chose an angle *θ* in the orthogonal plane space so that the difference between two consecutive angles is the golden number.^40^ This method is referred to as the spherical Fibonacci mapping. While it is not the optimal solution to the Thomson problem, its uniformity is rather good for our purposes (we have checked that its influence on the calculated properties is negligible) and the computational cost is lower. It also gives a convergence for values of *n* smaller than the random distribution methods described above, allowing us to use smaller sampling sizes, which is why we used this method for the rest of the surface sampling simulations presented in this article.

## 3 Results and discussion

### 3.1 Beyond Widom insertion

Widom insertion is a standard calculation method that consists in randomly inserting a single molecule inside an existing molecular system by randomly choosing its center and its rotation angle.^19^ By measuring the interaction energy of the molecule inserted, one can obtain the excess free energy Δ*F*_exc_ difference associated with its insertion into the framework, *i.e.*, the species chemical potential *μ*_i_. In the context of adsorption, this method has been used to randomly insert a molecule in the empty porous framework: after many cycles, the simulation has generated a diverse enough sample of points with different interaction energies *E*_int_, yielding the adsorption free energy Δ*F*_ads_ = −*RT* ln(〈exp(−*E*_int_/*RT*)〉), the Henry constant *K*_H_, and the adsorption enthalpy, Δ*H*_ads_ (eqn (1)), which has the opposite sign of the zero-loading isosteric heat of adsorption *Q*^0^_st_. The Henry constant^18,41,42^*K*_H_ (in mol kg^−1^ Pa^−1^) associated with the adsorption inside a crystalline framework of mass density *ρ*_f_ at temperature *T* can be derived using the following eqn (2):2
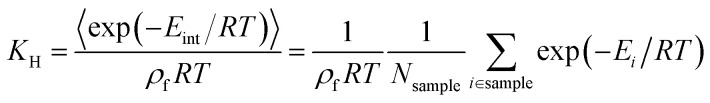
where *i* represents the point of a sample used in practice to calculate the integral.

If the free volume has been thoroughly explored, the Boltzmann average of the host/guest interaction energies converges to the adsorption enthalpy at infinite dilution. The Widom insertion method is very accurate, meaning that it converges to the “perfect” value of the adsorption enthalpy—for a given choice of interaction parameters—in the limit of infinite sampling. However, it is computationally expensive, and a lot of computational resources are wasted during this sampling in the calculation of interaction energies that have a negligible contribution to the overall Boltzmann average (points of high energy). Therefore, we can improve this method if we manage to sample preferentially points with the most negative interaction energies. To achieve this improvement we need to identify the characteristics of the adsorption sites that will have the highest weight (“count the most”) in the final average, while avoiding sampling parts of space where points will have marginal contribution.

The Voronoi sampling and the surface sampling presented in this article are examples of biased sampling methods, that follow this idea. The change in the sampling technique can dramatically improve the computation time required, and even a slight improvement in computational efficiency can have a non-negligible impact when dealing with datasets containing thousands of structures. This article focuses on biased sampling techniques to speed up adsorption enthalpy calculations. As a proof of concept, we only consider monoatomic adsorbates (in our tests, we used xenon) or adsorbates that can be modeled as a sphere (which is common in molecular simulations of species such as CH_4_). However, the methodology can be adapted to rigid polyatomic adsorbates, where the algorithm would need to be adapted by sampling the rotational degree of freedom of the adsorbed molecule.

In all the comparisons in this paper, we chose to take a Widom insertion simulation with 100 000 cycles (a very large number) as a ground truth or reference for the adsorption enthalpy values of every structure of the CoRE MOF 2019 database.^22^

### 3.2 Voronoi sampling

The use of the Voronoi decomposition of the pore space of materials for their geometric characterization has been widely employed in computational studies in the last decade,^21^ in particular since it was made easily available as part of the Zeo++ software package.^43^ Its use was extended recently to implement a novel sampling scheme, in a study proposing the ML-assisted screening of nanoporous materials for xenon/krypton separation. In this article, Simon *et al.*^14^ relied on a Voronoi tessellation of the nanoporous materials and assigned the potential adsorption sites (*i.e.*, the sampling points) at the nodes of this decomposition. The Voronoi tessellation identifies the vertices of polygons that correspond to the closest regions of each atom of the structure. These vertices (or Voronoi nodes) are the points equidistant to at least four atoms of the structure, and they can be associated with adsorption sites since they are positioned near the center of the pores. It is possible to calculate the host/guest interaction energies at every Voronoi node, and average them to obtain an approximation of the adsorption enthalpy. However, this sampling assumes that the nodes are close to the real, most favorable, adsorption sites. Or to put it differently, the adsorption sites need to be at the center of the pores, which is only true for structures with pore sizes close to the adsorbate size.

**Fig. 1:**
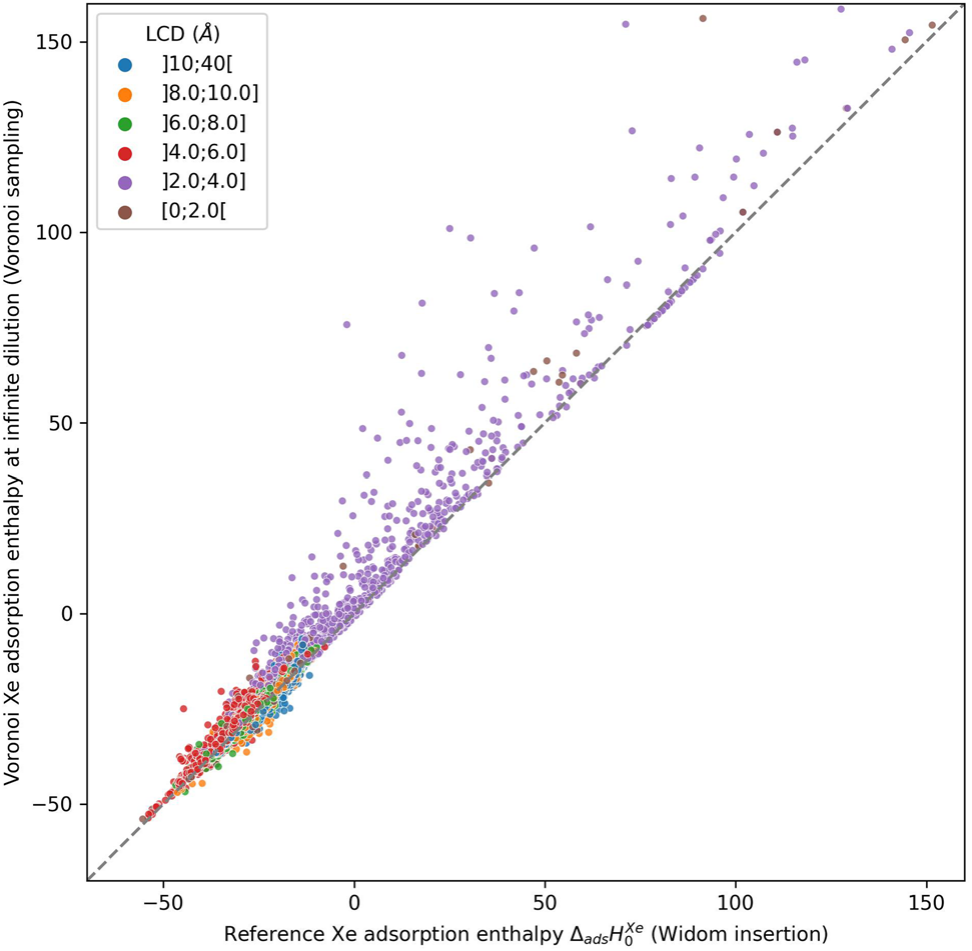
Scatter-plot of the enthalpies calculated by a Voronoi sampling compared to the enthalpies calculated by a 100k-step Widom insertion simulation of xenon in the structures of CoRE MOF 2019. The points are labeled according to the largest cavity diameter (LCD) belonging to one of the intervals.

To check the accuracy of this sampling technique, we compared it to our reference sampling, the Widom insertion with 100 000 cycles. Fig. 1 compares the enthalpy computed in the Voronoi sampling with the reference adsorption enthalpy (ground truth)—showing at the same time the largest cavity diameter for each porous framework. The correlation between the values of enthalpy is very good only for a restricted number of structures with enthalpy of around −50 kJ mol^−1^. For structures with higher enthalpy, the correlation starts to degrade, and becomes very poor for small-pore structures. For the points in purple, the largest cavity diameter is lower than the kinetic diameter of a xenon, where the sampling of the Voronoi nodes is clearly insufficient. In addition, the accuracy loss at the other points (larger pores) can be explained by the fact that the pores are slightly bigger and the center of the pore is not a good approximation of adsorption site position anymore: the adsorption sites are actually closer to the pore surface than to the center of the pore. This conclusion is what prompted us to propose a new sampling scheme based on the molecular surface of the pore space, which we will detail in the next sections.

The root mean square error (RMSE) and the mean absolute error (MAE) for Voronoi sampling are respectively 6.78 kJ mol^−1^ and 2.01 kJ mol^−1^, if we consider all structures in our set, which seem too high to be useful for screening purposes. However, non-porous materials would be screened out *a priori* in any high-throughput workflow, as they would not be of interest. We can only consider the structures with large enough cavities, larger than 3.7 Å (a bit lower than 3.96 Å Xe kinetic diameter). Thereby, the RMSE and MAE drop respectively to 2.11 kJ mol^−1^ and 1.55 kJ mol^−1^, which can be considered acceptable for a quick estimation of the guest–host affinity, but not for an accurate adsorption enthalpy calculation.

This is reinforced by the very low computational cost of the method. The Voronoi tessellation performed using the Zeo++ software is extremely quick and can output the positions of the Voronoi nodes in 0.28 s (measured as an average over all the structures of the CoRE MOF 2019 database), on a typical workstation (a single Intel Xeon Platinum 8168 core at 2.7 GHz). While a simple Python code for the energy calculation took around 27 s per structure, we benchmarked that a C++ optimized implementation can perform the Voronoi sampling in around 0.4 s. We only need to remember that this method takes a few hundred milliseconds per structure, while a Widom insertion needs approximately hundreds of seconds per structure. A Voronoi sampling is therefore 2 to 3 orders of magnitude quicker than a full sampling of the pore space.

This preliminary study identified a fast method for adsorption enthalpy calculations that can be widely used in screening procedures, but has limited accuracy for quantitative prediction. It raised important questions on the importance of selecting sampling points within the pore space of materials, and we wanted to develop an intermediate technique that is both fast and accurate for the prediction of adsorption enthalpy. For this purpose, we developed and optimized a new sampling technique that focuses the sampling on the surface of the material, which is expected to make up for the main flaws of the Voronoi sampling.

### 3.3 Construction of a surface sampling algorithm

In this section we describe the development of our surface sampling algorithm, with the goal of being more accurate than Voronoi sampling and faster than Widom insertion. Our initial idea is based on a series of theoretical considerations: (1) the strong adsorption sites are near the surface of the material; (2) by changing the problem from 3D to 2D sampling we can reduce the complexity; and (3) the algorithm can scale with the number of unique atoms in the structure (and not with the size of the unit cell), which is efficient because many porous frameworks have high symmetry. The first consideration ensures that this method will be more accurate than a Voronoi sampling, and the last two made us think that a well-optimized code would be fast. To confirm these hypotheses, we will analyze both the accuracy and the speed of this new algorithm and compare them to those of existing methods.

#### 3.3.1 Initial implementation

We present here our initial implementation of the surface sampling algorithm, and its basic principles. This first implementation is a relatively basic one and already performs well compared to the other methods. In the next sections, we refine it with two additional features that will improve its accuracy and its speed.

**Fig. 2:**
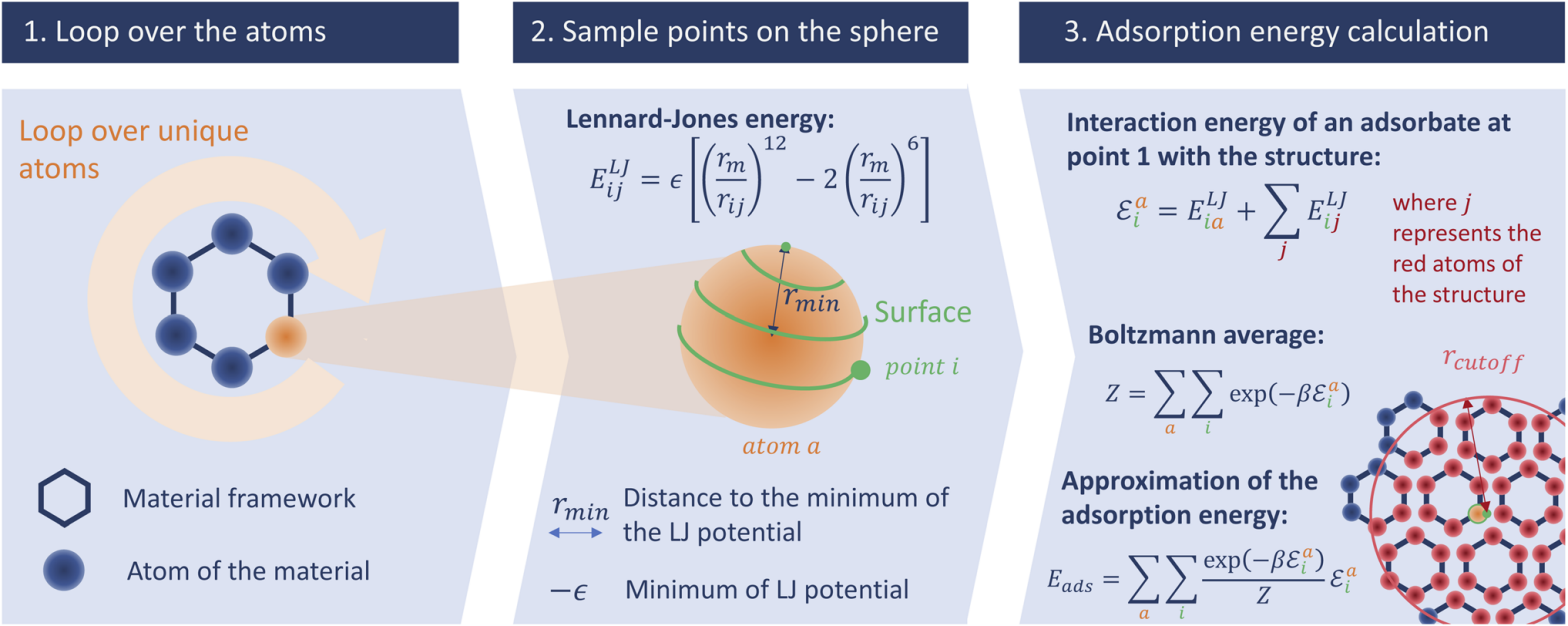
Schematic description of our surface sampling based on the three main steps of the algorithm: the loop over the unique atoms, the spiral sampling around each atom, and the energy averaging. The adsorbate is represented by the point *i* and is moved across all the points around the unique atoms of the structure.

This initial implementation speeds up the calculation of adsorption enthalpy in nanoporous materials by sampling interaction energies only near the surface. It is illustrated in Fig. 2. For this purpose, a loop over all unique atoms (as defined by crystalline symmetry) is performed. And for each atom, a sphere around its position is sampled using a uniform distribution around it; these points will be called sampling points and we can change the number of sampling points. The default radius chosen for the sampling spheres is the distance *r*_min_ = 2^1/6^*σ*_*ij*_ to the minimum of the LJ potential between atoms of type *i* (belonging to the framework) and *j* (the guest), corresponding to the strongest possible pair interaction (although the neighboring atoms will of course have an influence). After calculating the interaction energy at each of the sampled points, a Boltzmann average of these energies corresponds to a biased adsorption enthalpy, as described by eqn (1).

In order to validate the accuracy of the approximation made using this sampling, we applied this algorithm with 300 000 sampling points per unique atom. The results are illustrated in Fig. S1 and S2 and Table S1 of the ESI.† There is a good numerical agreement with the reference calculations; the RMSE and MAE are only around 0.90 kJ mol^−1^ and 0.66 kJ mol^−1^ considering all the structures from the database. Moreover, there is no noticeable difference in RMSE when considering the structures with a pore size above 3.7 Å (as determined by the largest cavity diameter, or LCD). Unlike Voronoi sampling, this method gives a consistent accuracy across all the structures of the database with a lower error. The fact that the RMSE error is below 1 kJ mol^−1^ is quite promising, and validates our intuition that this new sampling technique can be an intermediate between to the two previous methods (Voronoi and Widom).

**Fig. 3:**
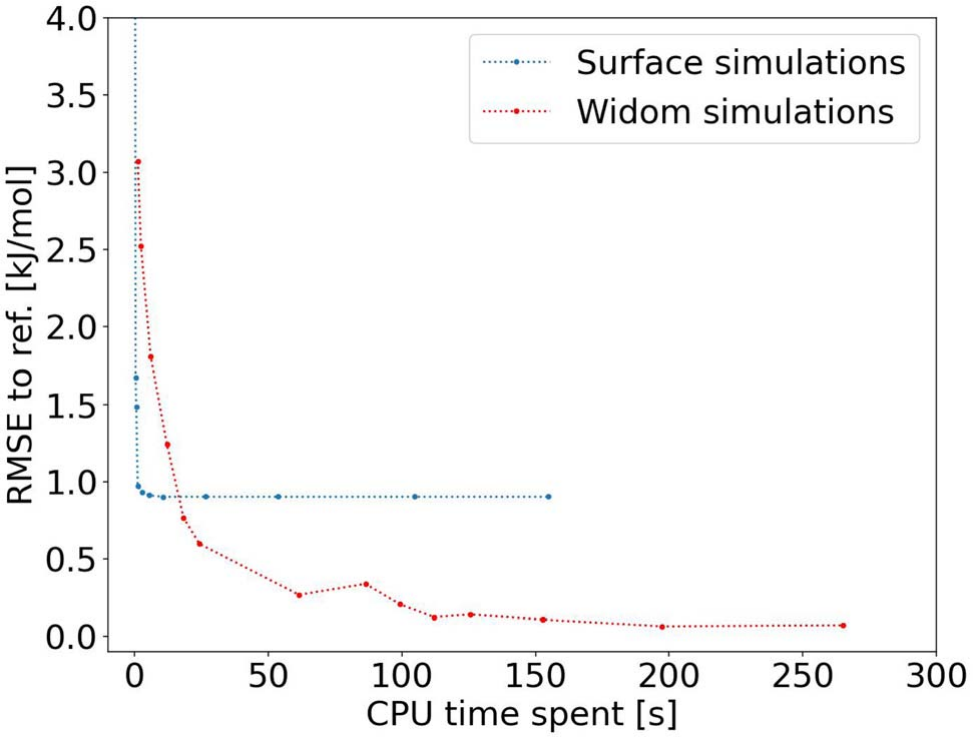
RMSE convergence of our algorithm (left) compared to a 100k-step Widom insertion simulation (right) for xenon adsorption in all the structures of the CoRE MOF 2019 database.

After proving the good accuracy of the method, we are now exploring the computation time required. We see in Fig. 3 that the method reaches an RMSE below 1.0 kJ mol^−1^ very quickly for an average CPU time of 1.2 s (Table S1†), corresponding to 2000 sampling points per atom. This is far less than the 150 s (Table S2†) required for a Widom insertion to reach its plateau value, for an RMSE of 0.10 kJ mol^−1^ with 12 000 cycles. Moreover, the Widom insertion needs around 14 s to reach a similar RMSE of 1.0 kJ mol^−1^, which is still slower than the surface sampling. We can conclude that this initial implementation of the surface sampling is faster than a standard Widom insertion, with a good accuracy.

However, this initial implementation of the method is slower than a Voronoi sampling that only needs to sample around 1600 points on average, instead of 13 000 sampled points on average (if we multiply by the average number of unique atoms). The sampling part would take approximately 0.15 s, and the Voronoi node generation 0.28 s, so our surface sampling algorithm remains 2 to 3 times slower (implemented in an identically compiled language, in this case C++). In order to improve the accuracy and performance, we have further tweaked the surface sampling method, adjusting the size of the sampling sphere and adopting a fast rejection criterion. The rejection of high-energy points with little contribution to the final enthalpy value can reduce the simulation time, whereas the size of the sampling sphere can improve the accuracy. The initially chosen sphere size only takes into account the interaction with the closest atom; we therefore chose to set it at the minimum of Lennard-Jones potential. However, the interaction with the neighboring atoms can further stabilize the adsorbate, so sampling further from this minimum could in consequence increase the accuracy of our surface sampling method.

#### 3.3.2 Size of the sampling sphere

The validity of the initial algorithm is based on the assumption that the adsorption site is at the minimum of the Lennard-Jones potential. It will only perform well if the closest atom contributes to almost all the interactions, but in real frameworks other neighboring atoms contribute to the host/guest interaction as well. We have found that in the vast majority of materials, the adsorption sites are located farther apart compared to the LJ potential minimum, in order to maximize the contribution of all atoms—and because of the dissymmetry of the interaction potential well. In order to see if this could be introduced in our algorithm, we implemented a parameter *λ*, and the sampling sphere radius is now defined using *R*_*λ*_ = *λσ*, where *σ* is the distance at which the LJ potential is zero. If *λ* = 2^1/6^, we fall back to our initial definition of the sampling sphere, and the adsorbent is at the minimum of the LJ potential of the atom. If *λ* = 1, the sampling sphere is at the zero of the LJ potential, and by increasing this parameter, we can check if our intuition was right.

**Fig. 4:**
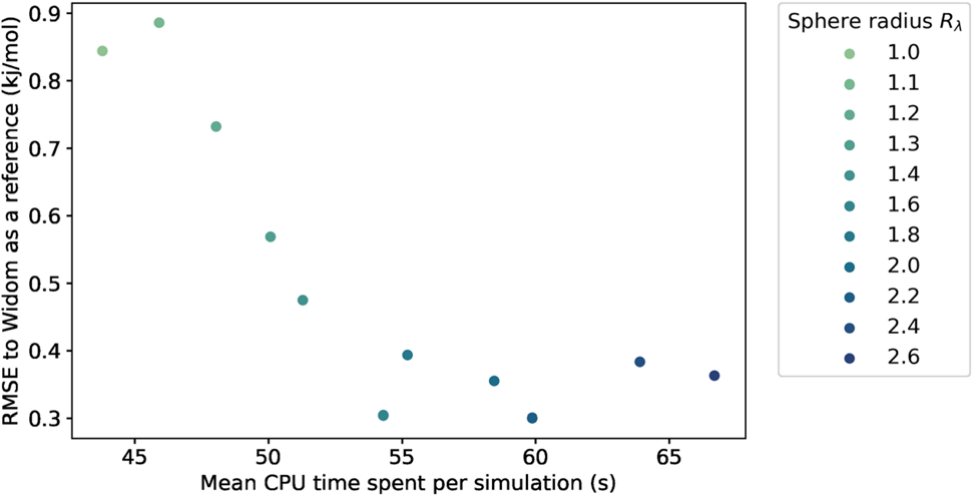
Influence of the sampling sphere radius *R*_*λ*_ on the average CPU time required for a simulation of 100k sampling points and the RMSE, compared to the reference adsorption enthalpy. The averaging is performed only on the structures with a largest cavity diameter (LCD) higher than 3.7 Å.

Because we have no physical model that would predict the optimal value of the sampling sphere, we followed a statistical approach. We studied the influence of the *λ* parameter on both the accuracy and the computation time, and the results are presented in Fig. 4. The RMSE turns out to be relatively high at around 0.90 kJ mol^−1^ for a radius of the sphere lower than the *r*_min_, and it then decreases for larger values of radius to reach a plateau at around 0.35 kJ mol^−1^. We confirm that by increasing the sampling sphere radius we can improve the accuracy of our algorithm, and find that for values of *λ* higher than 1.6, the accuracy is stabilized. We also find that increasing the sphere radius negatively impacts the computational efficiency, since it increases the number of neighbors considered in the energy calculation.

By choosing an optimal sampling sphere, we can more than halve the error, while increasing the computation time by around 20 percent, when comparing the case *λ* = 1.6 with *λ* = 1.1 (close to *r*_min_). In most cases, it will be an acceptable trade-off. However, in a case where the computation time is crucial, like in a rapid screening, the optimal choice might not be to increase the sampling sphere at *λ* = 1.6 but to have it lower at *λ* = 1.4 or *λ* = 1.2, and have an RMSE at around 0.5 kJ mol^−1^—still quite acceptable. The new scale parameter introduced in this section can therefore be tweaked to serve the users' purpose, whether it is to focus on the accuracy or to optimize the computation speed. If one wants to use it on a completely different database under very different conditions, then one can either choose a default value that works fine (*e.g. λ* = 1.4) or one can optimize the parameter on a small diverse sample of the unseen data.

#### 3.3.3 Rejection conditions

As shown above, our algorithm has better accuracy than Voronoi sampling, but its initial implementation was several times slower, which could make it unsuitable for screening applications in high-throughput workflows, where the number of structures to be screened can reach one million or more. To reduce the computational expense, we thought of rejecting the points with little contribution to the final enthalpy, *i.e.*, the largely positive interaction energies that would vanish in the exponential of the Boltzmann average.

**Fig. 5:**
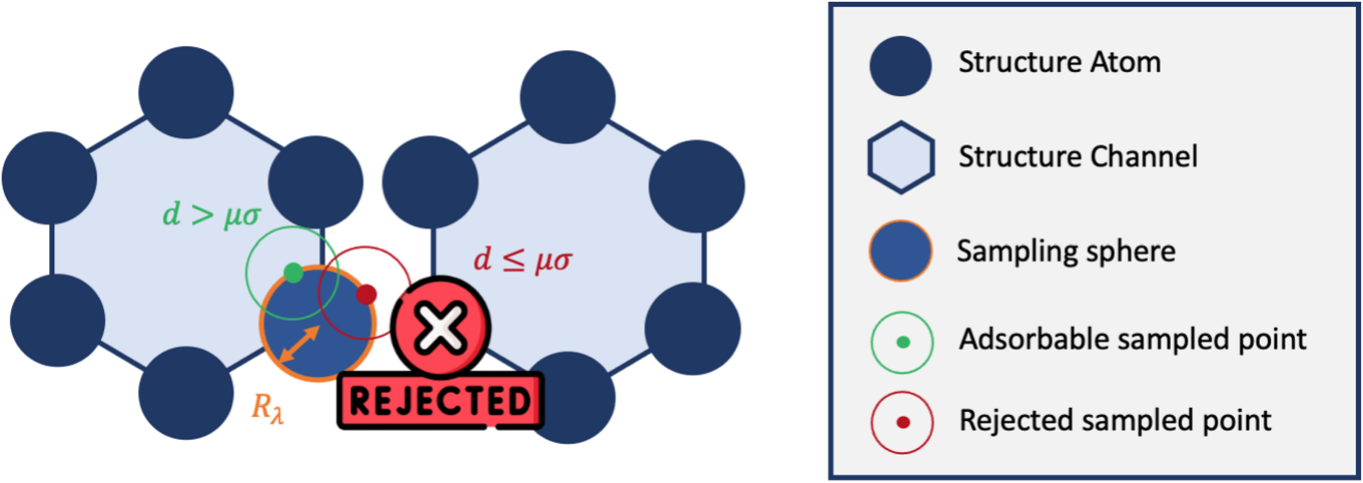
Simplified 2D representation of the principle of rejection conditions and the concept of sampling spheres inside the one-dimensional channels of a nanoporous material.

Inspired by typical methods for accessible surface calculations, we implemented a hard sphere rejection condition based on the distance to neighbors. If the adsorbate is too close to another atom of the structure, the sampling point is rejected, *i.e.*, its energy is not calculated (or considered to be infinite). We based this distance threshold on the *σ*_*ij*_ parameter of the Lennard-Jones potential. To determine the optimal threshold, we introduced a factor *μ* with real values between 0 and 1 that changes the size of the hard sphere rejection condition. If the guest–host distance is lower than *d*_*μ*_ = *μ* × *σ*, then the point is rejected. If *μ* = 0, then there is no rejection condition. And if *μ* = 1, we reject all points with a positive energy interaction with at least one atom of the structure. This condition could be a bit strong and points with non negligible contribution would end up being rejected. This rejection condition is schematically represented in Fig. 5.

**Fig. 6:**
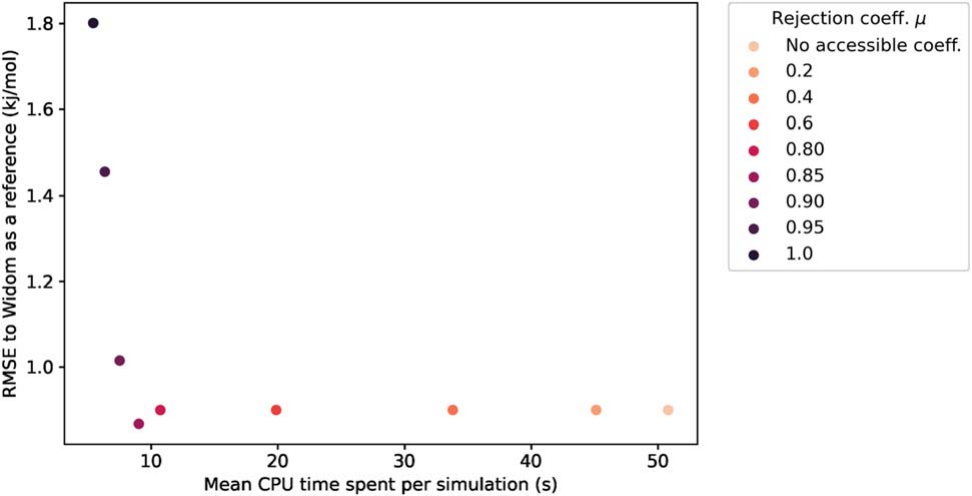
Influence of the rejection coefficient *μ* on the average CPU time required for a simulation of 100k sampling points and the RMSE compared to the reference adsorption enthalpy. The averaging is performed only on the structures with a largest cavity diameter (LCD) greater than 3.7 Å.

This rejection condition is expected to speed up the calculations, since the energy calculation is avoided for the rejected sampling points. The energy calculation accounts for the largest portion of the CPU time spent on the surface sampling. For the structure KAXQIL,^44^ the Lennard-Jones potential calculation represents up to 90% of the calculation time for 100 000 sampling points per sphere (with the initial algorithm). The higher the factor *μ*, the more rejections there would be. But, if too many points are rejected, the accuracy will drop. Here again, we used a statistical analysis to determine the optimal value of *μ*, making our sampling faster without compromising the accuracy of the enthalpy calculation. The results are displayed in Fig. 6.

The values of RMSE and time in Fig. 6 are averaged only on the most interesting structures for xenon adsorption (LCD ≥ 3.7 Å). For *μ* ≤ 0.85, increasing the value of *μ* improves the speed of the calculation without changing the RMSE.‡‡In fact, what we observe is a deterioration of the accuracy for structures with small pores because the probability of rejection in a confined space is really high and all sampled points end up rejected. But these points are not considered, if we apply the condition on the cavity size (LCD ≥ 3.7 Å). For high values of *μ*, the rejection condition is too strong and we reject points with non-negligible contribution to the overall enthalpy. The RMSE increases as a consequence. If we want to keep the accuracy unchanged, the optimal value is therefore *μ* ≃ 0.85, because it gives the lowest computation time with a similar RMSE. We note that it would be possible, in specific cases, to explore higher values of *μ* that trade a bit more accuracy in exchange of further speed gains.

For the simulations considered in Fig. 6, the use of a rejection condition *μ* = 0.85 makes the simulation four times faster than the standard algorithm. As we will see in the next section, the combination of optimal values for the *λ* and *μ* parameters generates an algorithm with very interesting performance compared to Voronoi sampling or Widom insertion.

### 3.4 Final surface sampling algorithm

#### 3.4.1 Performance comparison

**Fig. 7:**
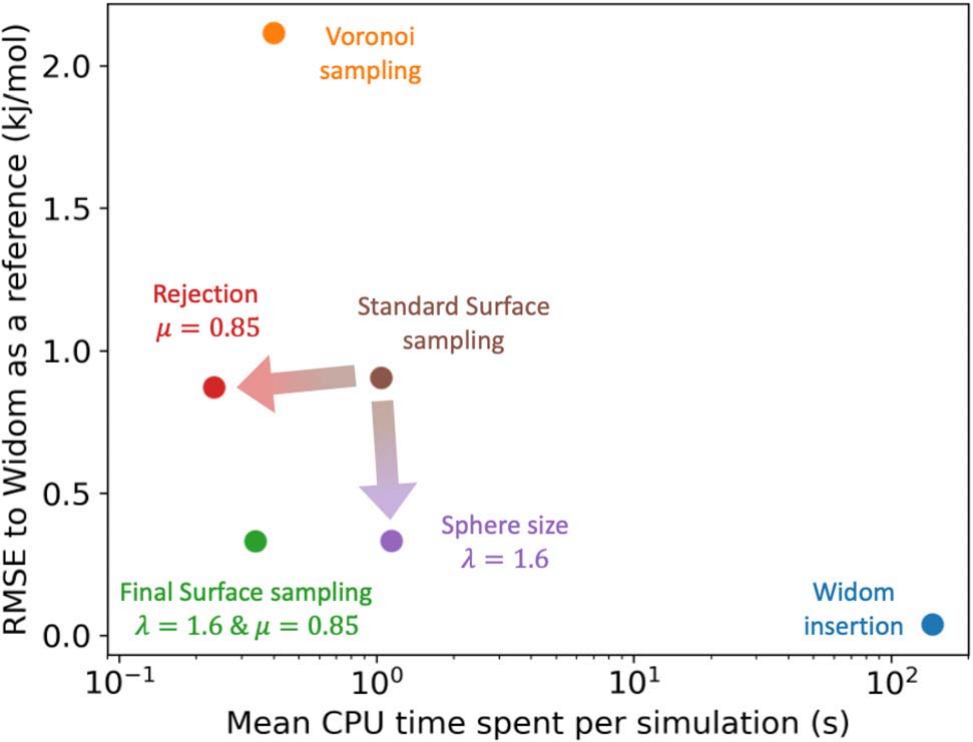
Comparison of the RMSE to the reference Widom insertion (100k cycles) and the average computation time for different types of enthalpy calculation methods. The surface sampling calculations were all performed with 2k sampling points on each sphere and the Widom simulations were performed using 12k cycles. These values correspond to the value at the convergence identified using Fig. 3.

For the calculation of adsorption enthalpy, our proposed surface sampling method is a good compromise between the accuracy of Widom insertion (full sampling of the porous space) and the speed of a less accurate method such as Voronoi sampling. The performance of our algorithm, including the two new features (sampling sphere scaling and rejection criterion) is illustrated in Fig. 7, where we can see the improvement brought about by each feature and how it compares to reference simulations. All CPU times are calculated using the smallest possible number of sampling points so that the respective algorithms reach convergence. With the implementation of a rejection condition, we find that surface sampling is even quicker than Voronoi sampling. Moreover, the increase in the size of the sampling sphere makes the surface sampling much more accurate, reaching an RMSE of 0.33 kJ mol^−1^ and an MAE of 0.21 kJ mol^−1^. The ideal set of parameters, determined for porous materials from the CoRE MOF 2019 database, is (*λ* = 1.6, *μ* = 0.85) in order to combine the lowest error and smallest computational cost. By combining both of these new features into the algorithm, we have a final surface sampling method with an RMSE of 0.33 kJ mol^−1^ and an average computation time of 0.34 s per structure. According to the data in Table S3,† it is about 6 times more accurate and 26% faster than Voronoi sampling, and it is also about 430 times faster than a Widom insertion with 12k cycles.

Finally, we suggest that the values of the parameters optimized in this work might need adjustment when applied to other adsorption systems. The optimal *μ* parameter depends on the size of the adsorbent, and it should be tweaked differently when considering another adsorbent. For instance, the set of structures used for the optimization of *μ* depends on the size of their cavities, and the 3.7 Å threshold chosen here would need to be changed according to the kinetic diameter of the adsorbate. Furthermore, as aforementioned in the section on rejection conditions, it is possible to trade-off a bit of accuracy for faster simulations especially in high-throughput screenings where speed is extremely important. Similarly, in the case of xenon, the cost of increasing the sphere size is around 10 to 20%. On very large databases, one could consider that this increase in the required computational time is not worth the accuracy improvement, and one could decide to keep a smaller sampling sphere. If this method is transposed to different molecular systems, its parameters should be tested on the specific database and adsorbate of interest.

#### 3.4.2 Calculation of the Henry constant and surface area

The main goal of our sampling algorithm is to calculate adsorption enthalpy at the zero-loading limit. But the method can also calculate the Henry constant and surface area of the materials at the same time, without significant additional computational cost. The Henry constant is a key metric for assessing the affinity of an adsorbate to a nanoporous structure. The A/B gas selectivity at low pressure is defined as a ratio of the Henry constants of components A and B. This important property can be calculated using eqn (2) in a Widom insertion calculation. Instead of using the interaction energies at the Widom inserted points, we can now use the surface sampled points to get an approximate value for the Henry constant.

Using the optimized set of parameters for surface sampling, we assessed the performance of our algorithm on the values of the Henry constant by comparing them to ground truth obtained by 100 000 cycles of Widom insertion. Since the Henry constant corresponds to the exponential of an adsorption free energy and we are more interested in the precision of the free energy, we are using a log-scale evaluation metric. For surface sampling, the log-RMSE of *K*_H_ is equal to 0.2, which means that the order of magnitude of the values is well predicted (Table S4†). If we consider the derived free energy Δ*F*_ads_ = −*RT* log(*ρ*_f_*RTK*_H_), the RMSE is of the order of 1.1 kJ mol^−1^ reached in about 1 s (Table S6†), whereas for Widom insertion, this level of error is also reached in a similar amount of time and 0.1 kJ mol^−1^ of RMSE is reached in about 86 s (Table S7†). For free energy calculations, surface sampling is still 86 times faster to converge. If we consider that the main target is the adsorption enthalpy, the Henry constant can be calculated with little additional computational cost and with reasonable accuracy: we get two thermodynamic properties of interest for the price of one.

The same goes for the determination of the surface area. We can adapt our algorithm to count the number of points of the sampling spheres that have a negative energy. These represent the points where a guest molecule can favorably interact; therefore when dividing it by the number of sampled points, we obtain a proportion of the adsorbable area of the sphere. Summing this over all atoms, we obtain the total surface area. This implementation is summed up in eqn (3):3
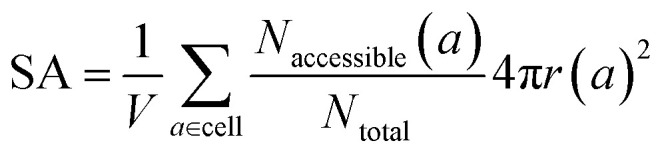
where *V* is the volume of the cell; *a* is the number of atoms of the cell; *N*_accessible_(*a*) is the number of accessible points around the atom *a*; *N*_total_ is the total number of sampling points; *r*(*a*) is the radius of the sampling sphere around the atom *a*. When we set *λ* = 1, we are sampling spheres that have a radius of *σ* and it is equivalent to considering hard spheres all defined using *σ* (convention used by RASPA2 to calculate surface areas). If we compare simulation with *λ* = 1, we obtain surface areas that are very close to the one obtained by RASPA2 (see Fig. S11 in the ESI†). However, when we consider *λ* = 1.6, we lose the accordance previously obtained and the points are weakly correlated at the log-scale (see Fig. S10 in the ESI†). The difference can be explained by the fact that the sphere size is larger, but the proportion of adsorbable points also changes. The relationship between these two adsorption surface areas is not trivial at all. Since the calculation of surface areas is quite cheap, this implementation would not be very useful, except for having a rough idea of the surface area.

## 4 Conclusions and perspectives

In the present article, we described a novel algorithm for the high-speed calculation of adsorption enthalpy in nanoporous materials that takes a unique approach to reduce the sampling necessary. This new algorithm is based on the core principle of dimensional reduction, from a volume problem to a surface one. The algorithm is proven to be significantly faster than the reference Widom insertion (random sampling of porous space). Moreover, the error associated is found to be in the order of 0.4 kJ mol^−1^, tested throughout the entire CoRE MOF 2019 database, for xenon adsorption. Even when compared to existing very fast sampling techniques such as Voronoi sampling, this surface sampling technique requires similar CPU time, combined with a better accuracy.

Based on these results, this algorithm has important potential for applications in the current computational analysis workflows of material databases, such as high-throughput screening studies. For instance, this algorithm can be used to get a fast approximation of the low-loading adsorption enthalpy of a molecule inside nanoporous materials. This cheap evaluation of enthalpy can be used to screen out the structures with little affinity with the targeted adsorbate molecule. It can also be used as a thermodynamic descriptor for selectivity prediction in a machine learning model, as performed by Simon *et al.*^14^ The computational speed-up brought about by this novel methodology can also enable the screening of materials databases at a larger scale in the future.

We note, moreover, that the speed of our method resides in the sampling technique itself, rather than in the actual energy calculation. While we have benchmarked it in this work for a simple Lennard-Jones interaction potential, this sampling technique could equally be used to speed up samplings of space based on more expensive modeling strategies, including polarizable force fields or density functional theory (DFT) calculations. In the literature, the need for cheap *ab initio* grade thermodynamic properties is usually fulfilled by using an importance sampling method based on a classical force field.^45^ In our method, the description of surface sampling is independent of any force field, and the sampling spheres can be defined according to kinetic radius, van der Waals radius or any other physically relevant distance. Consequently, given a definition of atomic radii, it is possible to define a surface on which to carry out other types of simulations such as neural network potential, DFT or any other force fields. Although the accuracy or relevance of such a sampling remains an open question, the approach will undeniably speed up the simulations. This could even be applied to calculate adsorption enthalpies while considering intrinsic structure flexibility,^46^ a task whose main drawback is the high computation time required. Since surface sampling is hundreds of time faster than standard methodologies, we could use hundreds of snapshots in a flexibility-aware calculation.

Finally, although the algorithm in its present form can already be applied in a wide range of applications, additional development work could allow us to generalize it to polyatomic adsorbates. For instance, we would need to work on a definition of the molecular radius for nonspherical adsorbates as well as all the orientation conformations of the adsorbent. We could imagine making the distance to the surface depend on the orientation of the adsorbate or sample a band volume on the surface. Although the best implementation of surface sampling for polyatomic adsorbates remains an open question, in theory it should be possible to apply it to more complex adsorbates than spherical noble gas. This would add more complexity to the algorithm but would not change the fundamental speed up due to surface sampling, since these orientation moves are also performed in other standard methodologies. To improve the accuracy even more, we could test hybrid samplings with multiple sampling spheres, or a combination of Voronoi nodes and sampling spheres. Another idea could be to have fractions of spheres that are oriented toward the center of pores given by the Voronoi node. In theory, having a wider variety of sampling points can only improve the sampling. There are therefore multiple possible sampling techniques that could be built around the method introduced herein. The code is made freely available on the group's GitHub, where further development will be released.

## Data availability

Data and code related to this study are available from our group repository at https://github.com/fxcoudert/citable-data. The RAESS code is available at https://github.com/coudertlab/RAESS.

## Author contributions

Both authors designed the study, analysed the results, wrote and revised the article. E. R. wrote the RAESS software and ran the molecular simulations.

## Conflicts of interest

There are no conflicts to declare.

## Supplementary Material

SC-014-D2SC05810C-s001
