# Supplementary material for: Rapid adsorption enthalpy surface sampling (RAESS) to characterize nanoporous materials
Source: Chem Sci. 2023 Jan 17;14(7):1797–807. doi: 10.1039/d2sc05810c (PMC9931060; doi:10.1039/d2sc05810c)
Supplement: SC-014-D2SC05810C-s001 [file SC-014-D2SC05810C-s001.pdf]

Electronic Supplementary Material (ESI) for Chemical Science.
This journal is © The Royal Society of Chemistry 2023

Supporting Information for: Rapid Adsorption Enthalpy Surface Sampling (RAESS) to Characterize Nanoporous Materials

Emmanuel Ren[†,‡] and François-Xavier Coudert[*,‡]

†*CEA, DES, ISEC, DMRC, Univ. Montpellier, Marcoule, France*

‡*Chimie ParisTech, PSL University, CNRS, Institut de Recherche de Chimie Paris, 75005 Paris, France*

E-mail: fx.coudert@chimieparistech.psl.eu

S1 Adsorption enthalpy

The adsorption enthalpy values of structures from CoRE MOF 2019 have been calculated using Widom insertion and the RAESS algorithm. In this section, we first give a visual comparison through some scatter-plots. We can visually see the improvement from the initial algorithm to the final algorithm. The raw data of the convergence plots are also shown to highlight the simulation time needed to reach convergence. Finally, the data of the performance plot that compares every enthalpy calculation methods (Widom insertion, Voronoi node sampling, RAESS).

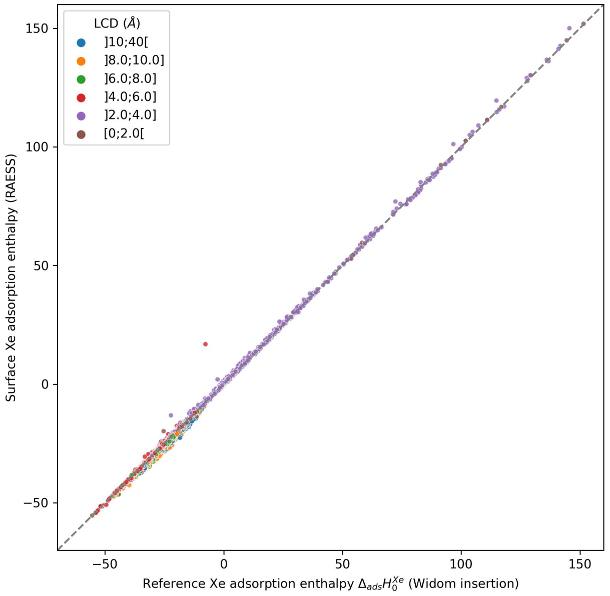

Figure S1: Scatter-plot of the enthalpies calculated by our initial algorithm ($\lambda = 2^{1/6}$ and $\mu = 0$) compared to the enthalpies calculated by a 100k step Widom insertion simulation of xenon in structures of CoRE MOF 2019 at 298 K.

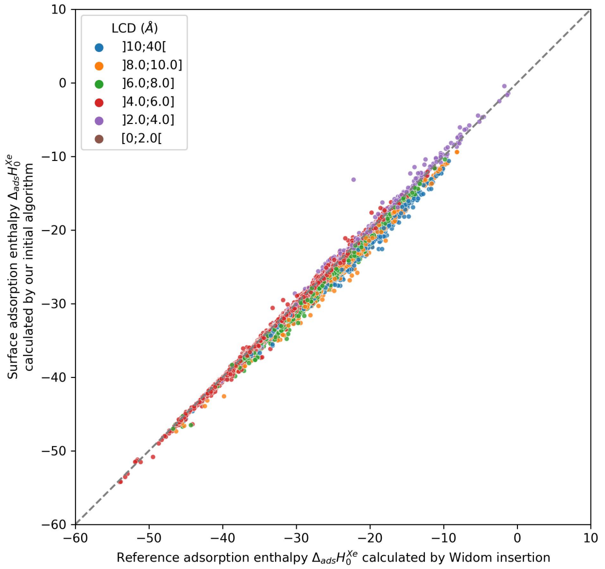

Figure S2: Scatter-plot of the enthalpies calculated by our initial algorithm ($\lambda = 2^{1/6}$ and $\mu = 0$) compared to the enthalpies calculated by a 100k step Widom insertion simulation of xenon in structures of CoRE MOF 2019 with LCD $\geq 3.7\,\text{Å}$ at 298 K.

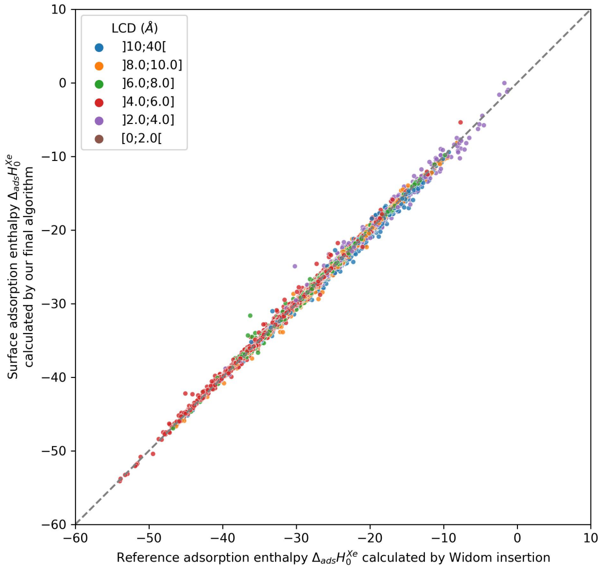

Figure S3: Scatter-plot of the enthalpies calculated by our final algorithm ($\lambda = 1.6$ and $\mu = 0.85$) compared to the enthalpies calculated by a 100k step Widom insertion simulation of xenon in structures of CoRE MOF 2019 with LCD $\geq 3.7\,\text{Å}$ at 298 K.

Table S1: Convergence plot raw data for **surface sampling** on **adsorption enthalpy**. In red the point considered at convergence.

RMSE_to_widom_100k	CPU_time (s)	step
6.419741	0.228990	100
4.464392	0.507070	150
4.294890	0.268637	200
1.673242	0.418563	500
1.485142	0.676902	1000
0.973481	1.208826	2000
0.932958	2.786422	5000
0.911168	5.440001	10000
0.901650	10.599231	20000
0.902108	26.543961	50000
0.901997	53.637419	100000
0.901995	104.863919	200000
0.901996	154.805544	300000

Table S2: Convergence plot raw data for **Widom insertion** on **adsorption enthalpy**. In red the point considered at convergence.

RMSE_to_widom_100k	CPU_time (s)	step
3.071953	1.238836	100
2.524768	2.422194	200
1.810364	6.049577	500
1.241951	12.135611	1000
0.764997	18.358402	1500
0.596282	24.320557	2000
0.266560	61.527327	5000
0.337272	86.456161	7000
0.204607	99.359254	8000
0.122742	112.065419	9000
0.139955	125.557030	10000
0.107073	152.668527	12000
0.064535	197.281619	15000
0.070857	264.995789	20000

Table S3: Raw data of the performance of each sampling method

Method	RMSE (kJ/mol)	Time (s)
surface_standard_2k	0.903953	1.045269
surface_radius_2k	0.331715	1.145985
surface_rejection_2k	0.870635	0.234415
surface_final_2k	0.330454	0.339397
voronoi	2.114351	0.400000
widom_12k	0.037631	145.058516

S2 Influence of the temperature: 600K

The RAESS method relies on the higher weight of the strong sites close to the surface of the pores. If we increase the temperature, the less attractive sites would play an increasing role and the accuracy of the method would drop. To grasp this limitation of the RAESS algorithm at higher temperature, we compared the results of a screening over the CoREMOF 2019 database.

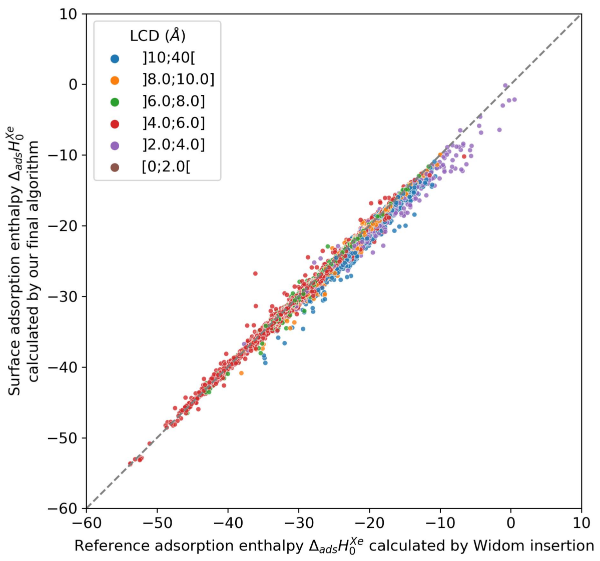

Figure S4: Scatter-plot of the enthalpies calculated by our final algorithm ($\lambda = 1.6$ and $\mu = 0.85$) compared to the enthalpies calculated by a 12k step Widom insertion simulation of xenon in structures of CoRE MOF 2019 with LCD ≥ 3.7 Å at 600 K.

The method is as expected less accurate but it still gives a reasonable correlation on the performance, with an RMSE of 0.70 kJ mol^{-1} and an MAE of 0.41 kJ mol^{-1}. The errors have almost doubled when going from 298 K to 600 K.

However, these limitations of the method are not crippling since adsorption processes are usually not performed at very high temperature. High temperatures are commonly used in temperature swing adsorption (TSA) to desorb the adsorbates rather than to adsorb them.

S3 Henry constant

S3.1 Derivation of the formula

Here we give a general derivation of the relation between the Henry constant K_H and the interaction potential $\mathcal{E}_\mathrm{int}$ of an adsorbate in a porous material. First, for an ideal gas the amount of adsorbed molecules n_ads can be expressed using the bulk density of the gas $\rho_\mathrm{ads,bulk}$ and the volume of the pores V_pore:

$$n_\mathrm{ads} = \rho_\mathrm{ads,bulk} \times V_\mathrm{pore} \tag{S1}$$

The pore volume can be seen as the continuous sum of each voxel times the Boltzmann probability of presence, which is represented by the following integral of the Boltzmann factors. This integral can then be changed to the average of the Boltzmann factors:

$$V_\mathrm{pore} = \int_V \exp(-\mathcal{E}_\mathrm{int}(\mathbf{r})/RT)d\mathbf{r} = V\langle \exp(-\mathcal{E}_\mathrm{int}/RT)\rangle \tag{S2}$$

If we apply the equation S2 and the ideal gas equation of state $P = \rho_\mathrm{ads,bulk}RT$ on the bulk gas in equilibrium, we can change the equation S1 to:

$$\frac{n_\mathrm{ads}}{V} = \frac{P}{RT}\langle \exp(-\mathcal{E}_\mathrm{int}/RT)\rangle \tag{S3}$$

If we now consider the gravimetric loading L (in $\mathrm{mol\,kg^{-1}}$), we need to divide the equation by mass density of the framework ρ_f:

$$L = \frac{n_\mathrm{ads}}{V\rho_f} = \frac{\langle \exp(-\mathcal{E}_\mathrm{int}/RT)\rangle}{\rho_f RT}P \tag{S4}$$

Since the Henry's law is described by $L = K_\mathrm{H} \times P$, we have the final equation:

$$K_\mathrm{H} = \frac{\langle \exp(-\mathcal{E}_\mathrm{int}/RT)\rangle}{\rho_f RT} \tag{S5}$$

S3.2 Data comparison

Here, we present the data on the Henry constants calculated by Widom insertion and the final RAESS algorithm. Their accuracy and time efficiency were compared thoroughly.

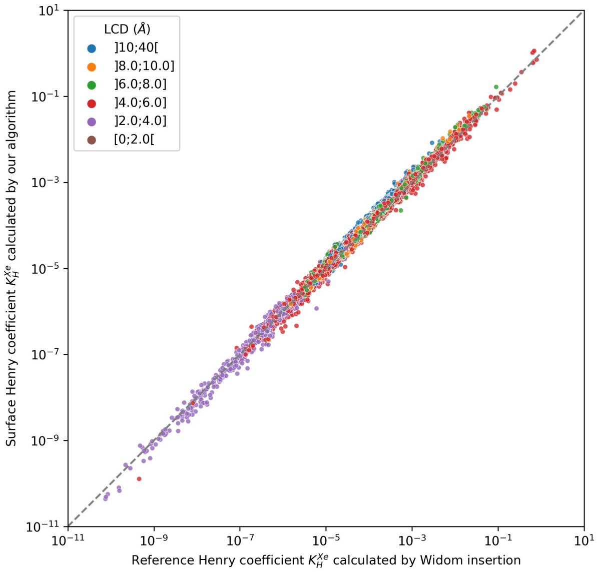

Figure S5: Scatter plot comparison of Henry constant calculated by our algorithm (300k sampling points) compared to the one calculated by Widom insertion (100k cycles).

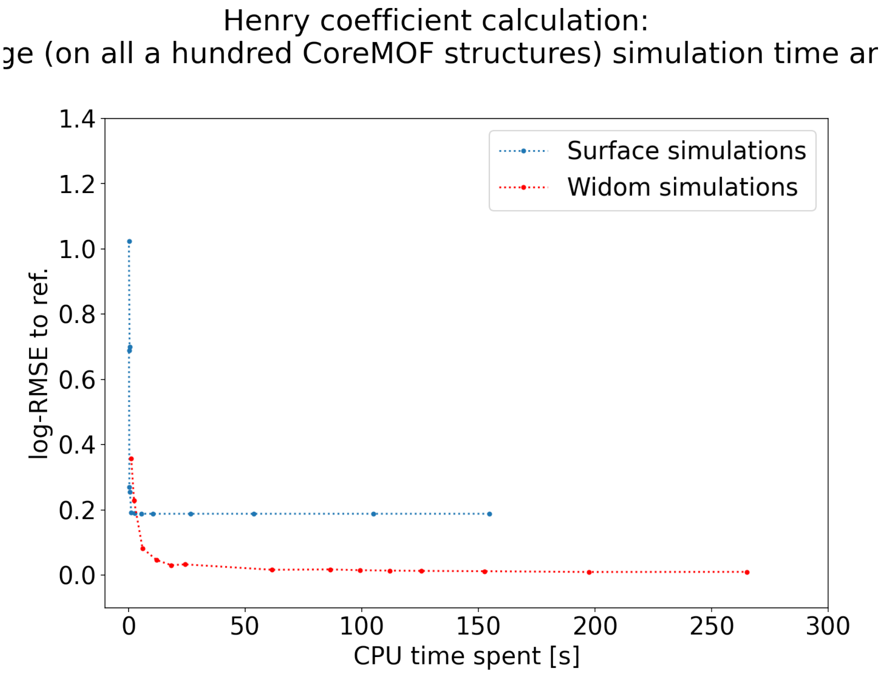

Figure S6: Convergence plot of the log-RMSE of the Henry constants.

Table S4: Convergence plot log-RMSE raw data for **surface sampling** on **Henry constants**.

log-RMSE_to_widom_100k	CPU_time (s)	step
1.024479	0.228990	100
0.699895	0.507070	150
0.689041	0.268637	200
0.270145	0.418563	500
0.254611	0.676902	1000
0.192593	1.208826	2000
0.190035	2.786422	5000
0.188364	5.440001	10000
0.188067	10.599231	20000
0.188177	26.543961	50000
0.188178	53.637419	100000
0.188178	104.863919	200000
0.188178	154.805544	300000

Table S5: Convergence plot log-RMSE raw data for **Widom insertion** on **Henry constants**.

log-RMSE_to_widom_100k	CPU_time (s)	step
0.358025	1.238836	100
0.228697	2.422194	200
0.082679	6.049577	500
0.046191	12.135611	1000
0.030677	18.358402	1500
0.033017	24.320557	2000
0.016464	61.527327	5000
0.017546	86.456161	7000
0.015203	99.359254	8000
0.013925	112.065419	9000
0.013096	125.557030	10000
0.012103	152.668527	12000
0.009682	197.281619	15000
0.010037	264.995789	20000

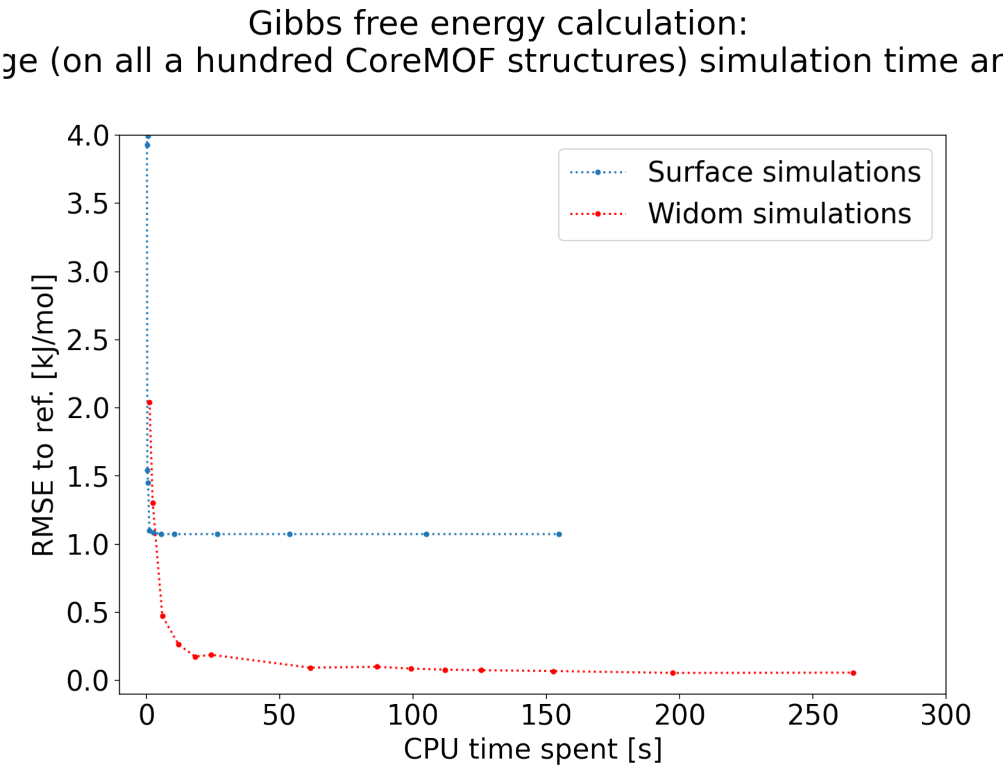

Figure S7: Convergence plot of the RMSE of Gibbs free energy calculated from Henry constants.

Table S6: Convergence plot RMSE raw data for **surface sampling** on **Gibbs free energy**. In red the point considered at convergence.

RMSE_to_widom_100k	CPU_time (s)	step
5.844794	0.228990	100
3.992996	0.507070	150
3.931076	0.268637	200
1.541217	0.418563	500
1.452590	0.676902	1000
1.098770	1.208826	2000
1.084176	2.786422	5000
1.074642	5.440001	10000
1.072951	10.599231	20000
1.073573	26.543961	50000
1.073580	53.637419	100000
1.073581	104.863919	200000
1.073582	154.805544	300000

Table S7: Convergence plot RMSE raw data for **Widom insertion** on the RMSE of **Gibbs free energy**. In red the point considered at convergence.

RMSE_to_widom_100k	CPU_time (s)	step
2.042582	1.238836	100
1.304748	2.422194	200
0.471696	6.049577	500
0.263529	12.135611	1000
0.175016	18.358402	1500
0.188365	24.320557	2000
0.093928	61.527327	5000
0.100103	86.456161	7000
0.086737	99.359254	8000
0.079443	112.065419	9000
0.074714	125.557030	10000
0.069047	152.668527	12000
0.055238	197.281619	15000
0.057265	264.995789	20000

S4 Xe/Kr Selectivity

Let us consider the following exchange equilibrium between the free gas and the adsorbed gas to model the competition between the xenon and krypton for adsorption sites.

$$\mathrm{Xe_{(g)}} + \mathrm{Kr_{(ads)}} \rightleftharpoons \mathrm{Xe_{(ads)}} + \mathrm{Kr_{(g)}} \tag{S6}$$

The selectivity can be seen as the equilibrium constant of the previous equation. At infinite dilution we can associate it with the Henry constants:

$$s_0^{\mathrm{Xe/Kr}} = \frac{n_{\mathrm{ads}}^{\mathrm{Xe}} P^{\mathrm{Kr}}}{n_{\mathrm{ads}}^{\mathrm{Kr}} P^{\mathrm{Xe}}} = \frac{K_{\mathrm{H}}^{\mathrm{Xe}} P^{\mathrm{Xe}} P^{\mathrm{Kr}}}{K_{\mathrm{H}}^{\mathrm{Kr}} P^{\mathrm{Kr}} P^{\mathrm{Xe}}} = \frac{K_{\mathrm{H}}^{\mathrm{Xe}}}{K_{\mathrm{H}}^{\mathrm{Kr}}} \tag{S7}$$

where P^{Xe} and P^{Kr} are the partial pressures, $K_{\mathrm{H}}^{\mathrm{Xe}}$ and $K_{\mathrm{H}}^{\mathrm{Kr}}$ are the Henry constants, $n_{\mathrm{ads}}^{\mathrm{Xe}}$ and $n_{\mathrm{ads}}^{\mathrm{Kr}}$ the quantity adsorbed of respectively xenon and krypton.

S4.1 Discussion on the rejection condition

To calculate a selectivity, the rejection condition on xenon can be high since we are interested in the most favorable materials for xenon adsorption. But for krypton, we want to accurately describe very low Henry constants, because a selective material would also be a material unfavorable to krypton. For these reasons, the μ parameter needs to be chosen wisely and needs to be low enough to have accurate selectivities.

Here, we give some values of the error on the selectivity depending on the rejection condition we chose.

Table S8: Effect of the rejection condition of the krypton surface simulation on the accuracy of the Xe/Kr selectivity.

rejection parameter μ	log10-RMSE to widom 100k	log10-MAE to widom 100k
0.85	0.107	0.077
0.50	0.0635	0.0402
0.20	0.0637	0.0403

According to the quick study here, the optimal value is $\mu = 0.5$ since it gives the best accuracy in a minimal amount of time.

S4.2 Correlation analysis for $\mu = 0.5$

The selectivity can be compared directly using the a log-scale plot and log-scale metrics. If we apply the log10 to the selectivities, we obtain RMSE of 0.064 and MAE of 0.04. This means that we have an error of about 0.06 when we compare orders of magnitude of the selectivity. For example, if a selectivity is predicted to be $s = 10^{-7}$, then s would be in the interval $[10^{-7.06}, 10^{-6.94}]$.

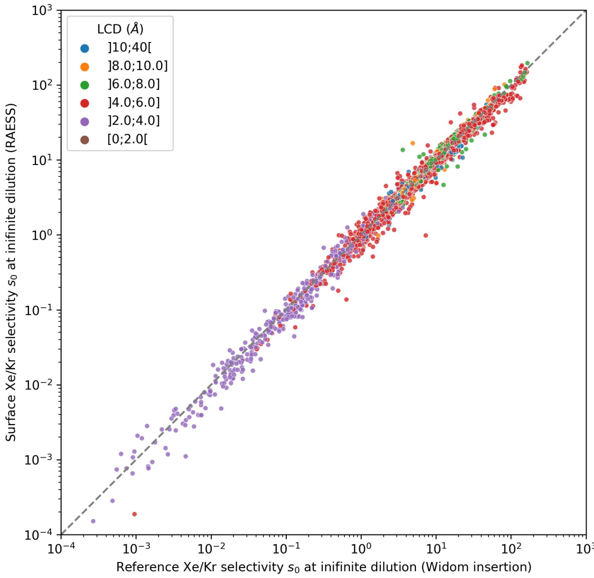

Figure S8: Scatter plot comparison of the selectivity calculated by the Widom insertion compared to the final implementation of RAESS (in log scale). log10-RMSE = 0.064 and log10-MAE = 0.040

To be able to give a thermodynamic interpretation, we can define an exchange Gibbs free energy associated to this selectivity:

$$\Delta_{\text{exch}} G_0^{\text{Xe/Kr}} = -RT \ln(s_0^{\text{Xe/Kr}}) \tag{S8}$$

Using this exchange Gibbs free energy, we can assess much more easily the performance

of the approach. The RMSE is about $0.36\,\mathrm{kJ\,mol^{-1}}$. We cannot compare it to the adsorption enthalpy errors, since the ranges and interpretation are very different. Here, the selective materials have a negative $\Delta_{\mathrm{exch}}G_0^{\mathrm{Xe/Kr}}$ and it goes to a maximum value of about $-12.7\,\mathrm{kJ\,mol^{-1}}$. The relative error is of course higher on the Gibbs free energy. This is due to a higher uncertainty on the Henry constant and to the denominator term brought by the krypton.

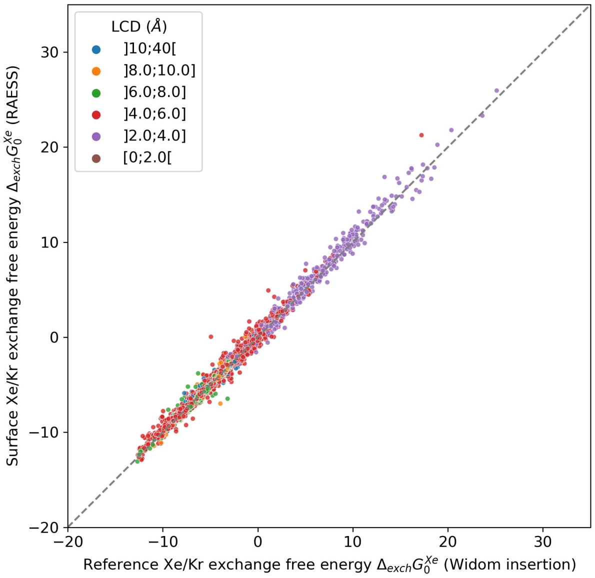

Figure S9: Scatter plot comparison of the exchange Gibbs free energy $\Delta_{\mathrm{exch}}G_0^{\mathrm{Xe/Kr}}$ calculated by the Widom insertion compared to the final implementation of RAESS (RMSE=$0.36\,\mathrm{kJ\,mol^{-1}}$ and MAE=$0.23\,\mathrm{kJ\,mol^{-1}}$).

If we want to know how well the RAESS algorithm would work in real situations, we tried to compare the top 100 most selective materials given by RAESS and a Widom simulation (RASPA2). We found that 83 structures of the top 100 given by RAESS are in the top 100 given by Widom insertion. As the correlation is not perfect, it is inevitable that there is a change in the order of the top 100 given by these two methods. This number of 83% proves that the difference is quite narrow. If we enlarge to the top 150 of the Widom simulation, 94 are present in the top 100 of the surface simulation. A vast majority of the best candidates given by the Widom insertion simulation are found by the RAESS algorithm.

S5 Surface area

Comparison of the surface areas calculated by RAESS and by RASPA2. In our algorithm, we sample at a further distance from the atoms than a surface area calculation algorithm would do. For this reason, the surface areas calculated by our algorithm are correlated to the ones given by RASPA2 by are very different.

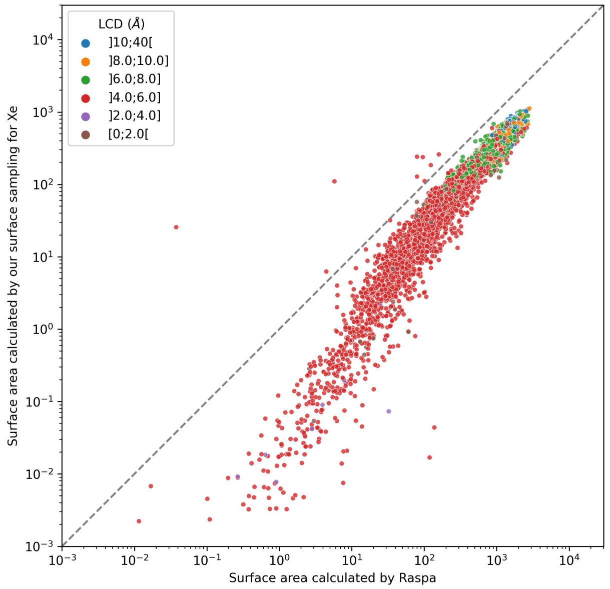

Figure S10: Scatter plot comparison of surface area calculated by our final algorithm (sampling at $1.6 \times \sigma$) and the ones calculated by Raspa with the same values of sigma ($m^2\,cm^{-3}$)

However, when modifying our code to match the standard surface sampling algorithm, we have a better agreement between both methods.

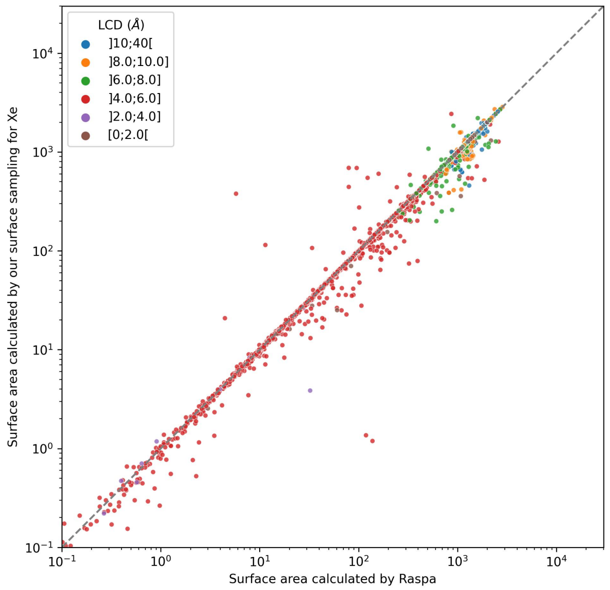

Figure S11: Scatter plot comparison of surface area calculated by a modified version of our algorithm (sampling at σ) and the ones calculated by Raspa with the same values of sigma ($\mathrm{m^2\,cm^{-3}}$)

S6 Other databases

S6.1 ToBaCCo

We randomly selected 1000 structures from the 13,511 structures of ToBaCCo to test the robustness of the RAESS method on other databases. Since the ToBaCCo database contains structures with larger pores as suggested by a Moosavi et al., there more errors on these materials that are unfavorable for small molecule (such as Xe) adsorption. The correlation is therefore much weaker than in the CoRE MOF 2019 database. The large pores are not very interesting for small molecule adsorption purposes. The errors should be nuanced by the relative irrelevance of these materials.

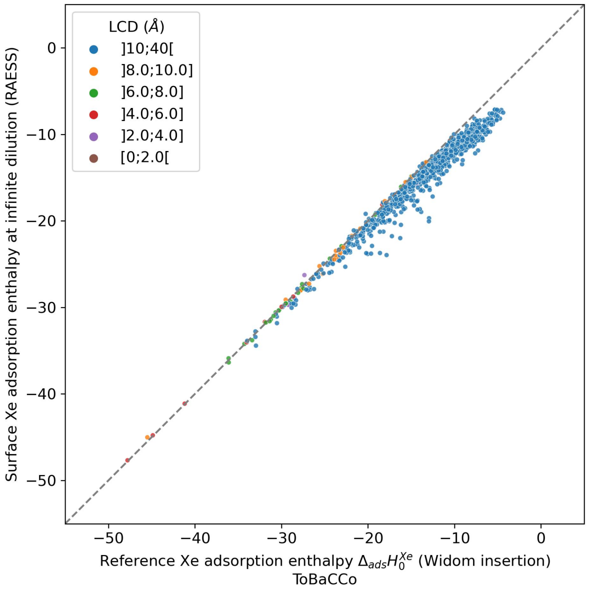

Figure S12: Scatter plot comparison of the xenon adsorption enthalpy calculated by the RAESS algorithm and the Widom insertion (RASPA2) on the ToBaCCo database. RMSE $= 1.79\,\mathrm{kJ\,mol^{-1}}$ and MAE $= 1.48\,\mathrm{kJ\,mol^{-1}}$. 915 structures have a LCD greater than 10 Å.

We can note that the points with weaker correlations correspond to the ones with an LCD greater than 10 Å.

S6.2 Amorphous materials

We tested our algorithm on the amorphous database and retrieved the top 20 materials for xenon (the whole csv file can be found on our group's GitHub). The Raspa software is not able to run on these amorphous structures with our computers, the memory is filled up and the job is killed (didn't investigate where this comes from). Therefore, there is no comparison point with a Widom simulation from Raspa.

Table S9: The 20 most xenon favorable amorphous materials according to the RAESS algorithm. The results on the whole amorphous database is given in csv format on the Github of the group.

Structure Name	$\Delta_{\text{ads}}H_0^{\text{Xe}}$ (kJ mol^{-1})	K_{H}^{Xe} ($\text{mol kg}^{-1}\,\text{Pa}^{-1}$)	CPU_time (s)
aCarbon-Marks-id035	-63.55	6.98e-01	285.45
aCarbon-Marks-id004	-60.14	1.50e-01	156.32
aCarbon-Marks-id031	-59.83	2.70e-01	297.07
aCarbon-Marks-id043	-59.65	1.39e-02	292.42
aCarbon-Marks-id039	-58.60	1.57e-03	295.48
aCarbon-Marks-id059	-58.53	4.39e-02	279.67
aCarbon-Marks-id034	-56.48	3.86e-01	250.71
aCarbon-Marks-id047	-56.41	1.04e-02	284.72
aCarbon-Marks-id023	-54.92	9.69e-03	282.40
aCarbon-Marks-id017	-54.87	1.06e-01	361.74
aCarbon-Marks-id030	-54.06	2.39e-01	254.46
aCarbon-Marks-id018	-53.82	1.49e-01	367.21
aCarbon-Marks-id008	-53.02	1.50e-01	202.25
aCarbon-Marks-id006	-53.01	1.39e-01	186.48
aCarbon-Marks-id005	-52.63	7.64e-02	167.30
aCarbon-Marks-id038	-52.43	1.53e-02	260.55
aCarbon-Marks-id026	-52.23	1.91e-03	237.32
aCarbon-Marks-id058	-51.69	3.23e-02	235.47
aCarbon-Marks-id007	-51.01	9.97e-02	192.23
aCarbon-Marks-id019	-50.61	8.60e-02	375.08
